# Spinal Cord Stimulator Paddle Lead Surgery Complicated by Cerebrospinal Fluid Leak and Fistula Formation

**DOI:** 10.7759/cureus.7619

**Published:** 2020-04-10

**Authors:** Namath S Hussain, Jorrdan N Bissell, Vadim Gospodarev, Adil Hussain

**Affiliations:** 1 Neurological Surgery, Loma Linda University Medical Center, Loma Linda, USA; 2 Neurological Surgery, Loma Linda University School of Medicine, Loma Linda, USA; 3 Physical Medicine and Rehabilitation, Rancho Los Amigos National Rehabilitation Center, Downey, USA

**Keywords:** spinal cord stimulation, intractable pain, spine surgery, pseudomeningocele, cerebrospinal fluid leak, cerebrospinal fluid fistula

## Abstract

Spinal cord stimulation (SCS) paddle leads placed via laminectomy procedures have become common as more data accumulates with regards to their clinical efficacy. In this paper, we describe a case of a 72-year-old male patient with failed back surgery syndrome (FBSS) who underwent a thoracic laminectomy for permanent paddle lead placement. He went on to develop a complication that resulted in a large cerebrospinal fluid leak with a cerebrospinal fluid fistula formation.

## Introduction

Spinal cord stimulation (SCS) is a modality used for treating chronic pain syndromes. It involves the delivery of high-dose electrical current to the spinal cord dorsal columns. This modality has been definitively shown to control pain, leading to proposals of multiple postulated mechanisms as to how the stimulation controls pain [1]. It is well documented that SCS is a safe intervention for the treatment of chronic pain syndromes. However, several studies have also reported complications with this surgical procedure [2-5]. As is the case with any neurosurgical procedure, it is important to consider risks associated with this treatment modality. The case presented describes a complication associated with SCS and a surgical technique that was used to bring about its resolution.

## Case presentation

**Figure 1:**
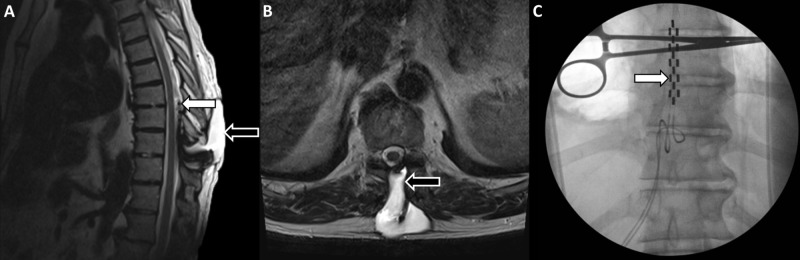
Imaging studies A: preoperative T2 sagittal MRI shows a pseudomeningocele (black arrow) and a spinal cord stimulator paddle (white arrow); B: preoperative T2 axial MRI redemonstrates the pseudomeningocele (black arrow); C: intraoperative fluoroscopic imaging shows the spinal cord stimulator paddle (white arrow) MRI: magnetic resonance imaging

A 72-year-old male with failed back surgery syndrome (FBSS) was initially evaluated and treated at an outside hospital. After a successful percutaneous trial lead placement, he underwent thoracic laminectomy for permanent paddle-lead placement. A few weeks after the surgery, he developed a growing painful lump at his thoracic incision site. On physical examination, it appeared to be a fluid-filled collection underneath the thoracic incision site, which seemed well-healed and not leaking. There were no signs of infection or erythema. Lab results were within normal limits. An MRI of the thoracic spine showed a large dorsal spinal fluid collection with a paddle lead floating in the middle of the large spinal fluid pocket (Figure 1A, 1B). Additionally, we utilized intraoperative fluoroscopic imaging to guide our surgical intervention (Figure 1C).

The patient underwent a thoracic laminectomy for the repair of the cerebrospinal fluid leak and fistula closure with the removal of implanted spinal cord stimulator and lumbar drain placement. During surgery, the paddle was found floating in the middle of a large extradural cerebrospinal fluid collection. Multiple old Prolene sutures (Ethicon Inc., Somerville, NJ) were identified surrounding a large dural defect, indicating an unsuccessful closure attempt in the past. The dural defect was closed with muscle onlay graft, Prolene sutures, and DuraSeal (Integra LifeSciences, Princeton, NJ), a polyethylene glycol hydrogel. A lumbar drain was placed and spinal fluid was drained for one week while the thoracic dural repair was healing. After one week of no leaking or fluid collection at the thoracic incision, the lumbar drain was removed, and the patient was discharged home a few days later. Nonabsorbable skin sutures were removed two weeks postoperatively. The patient continues to do well without any signs of cerebrospinal fluid leakage.

## Discussion

Pain and its effects on people are complex, multifaceted phenomena, which involve pain nociceptors in the body picking up stimuli that are interpreted as painful. These stimuli are sent through the dorsal columns of the spinal cord, up to the thalamus, and then the somatosensory cortex. As these signals travel along this pathway repeatedly, the strength of this connection solidifies, just as memory does, and can become chronic. Blocking these signals at this level, the dorsal columns initiate a pain control mechanism that can inhibit the actual sensation and body’s experience with pain. A spinal cord stimulator device is composed of a lead and a pulse generator, which are both implanted completely underneath the skin. The impulse produced by the leads effectively blocks the transmission of pain signaling in the dorsal column. Widespread research on the usage of SCS has led to a large body of literature that demonstrates the efficacy of SCS treatment [6-9]. Pain of various etiologies, such as FBSS, complex regional pain syndrome, radiculopathy, peripheral vascular disease, and diabetic neuropathy can be effectively managed with SCS treatment [10].

SCS treatment is not without risks and, therefore, is commonly used only after more conservative treatments have failed. Patient screening for contraindications is imperative. Prior to implantation, major points of patient screening include patient history and disease status, coagulative status, presence of a pacemaker or other medical devices, current infection and risk of infection development, and psychological screening [11]. Mechanical complications can occur and include lead fracture or disconnection, cerebrospinal fluid leak, lead migration, and implantable pulse generator failure [10]. Other complications reported include infection, pain at the implant site, implantable pulse generator seroma, epidural fibrosis, epidural hematoma, incidental durotomy, and, rarely, neurological injury. The occurrence of incidental durotomy with a cerebrospinal fluid leak is reported in 0.3-2% of patients and can usually be managed conservatively [10,12]. Complications must always be considered by the operating surgeon when deciding between percutaneous insertion of electrodes under fluoroscopy and performing a laminectomy for placement of paddles.

In our case, we were unable to determine when the original dural defect occurred, as it could have happened either during the percutaneous trial lead placement or when the patient underwent thoracic laminectomy for permanent paddle lead placement at the outside hospital. In rare cases when pseudomeningoceles do occur, dural repair, lumbar drain, or other cerebrospinal fluid leak intervention must be employed. Pseudomeningocele etiology is based on a dural opening, which leaks cerebrospinal fluid and subsequently forms a fluid-filled pocket. Pseudomeningoceles are extradural collections of cerebrospinal fluid that result from a dural defect and violation of subarachnoid space [13]. Sequelae of pseudomeningoceles include intracranial hypotension, site pain, nerve compression, external fistula tract formation, and meningitis [13]. A congenital dural defect, traumatic puncture, or iatrogenic durotomy are all possible causes of the pathologic dural opening found in pseudomeningoceles. Postoperative pseudomeningoceles are most likely caused by dural tears due to unintentional durotomy without complete closure or dural puncture by sharp bone edges post-laminectomy [13]. Complications of dural tears include spinal fluid leaks, post-dural puncture headache (PDPH), and meningitis [14]. Although pseudomeningoceles have been reported to resolve spontaneously, management is required for symptomatic patients [13]. This patient presented with pain and tenderness at the site of the pseudomeningocele. Along with the presence of the SCS lead displacement, a necessity for surgical management was indicated. There are various techniques to repair dural tears, which require evaluation of the clinical situation to determine the best approach for each case. For this case, a muscle onlay graft was utilized as an effective treatment approach.

## Conclusions

Although SCS has been a well-received adjunct for pain control in chronic pain patients, the placement of these mechanical devices is not without risk. Multiple studies have reported SCS as a safe procedure due to its reversibility and minimally invasive nature. We reported a case of a 72-year-old patient with FBSS who underwent a thoracic laminectomy for permanent paddle lead placement and unfortunately developed a pseudomeningocele. We described the details of surgical interventions that were employed to repair this spinal fluid leak, which involved the closure of the dural defect with a muscle onlay graft and placement of a lumbar drain, in order to elicit the best possible outcome in light of this particular SCS complication. This case illustrates how important it is to not understate the risks associated with this treatment modality. A thorough evaluation of the patient’s history can alert providers to potential risks or pitfalls. Attention to surgical details can reduce the rate of complications and improve outcomes in this patient population.
